# Clinicopathological features and prognosis of metastatic tumors in the small bowel: a large multicenter analysis of the JSCCR database in Japan

**DOI:** 10.1007/s00535-025-02322-z

**Published:** 2025-11-18

**Authors:** Akiyoshi Tsuboi, Shiro Oka, Takeshi Yamada, Keigo Mitsui, Hironori Yamamoto, Keiichi Takahashi, Akio Shiomi, Kinichi Hotta, Yoji Takeuchi, Toshio Kuwai, Fumio Ishida, Shin-Ei Kudo, Shoichi Saito, Masashi Ueno, Eiji Sunami, Tomoki Yamano, Michio Itabashi, Kazuo Ohtsuka, Yusuke Kinugasa, Takayuki Matsumoto, Tamotsu Sugai, Toshio Uraoka, Koichi Kurahara, Shigeki Yamaguchi, Tomohiro Kato, Masazumi Okajima, Hiroshi Kashida, Fumihiko Fujita, Hiroaki Ikematsu, Masaaki Ito, Motohiro Esaki, Masaya Kawai, Takashi Yao, Madoka Hamada, Takahiro Horimatsu, Keiji Koda, Yasumori Fukai, Koji Komori, Yusuke Saitoh, Yukihide Kanemitsu, Hiroyuki Takamaru, Kazutaka Yamada, Hiroaki Nozawa, Tetsuji Takayama, Kazutomo Togashi, Eiji Shinto, Takehiro Torisu, Akira Toyoshima, Naoki Ohmiya, Takeshi Kato, Eigo Otsuji, Shinji Nagata, Yojiro Hashiguchi, Kenichi Sugihara, Yoichi Ajioka, Shinji Tanaka

**Affiliations:** 1 Department of Gastroenterology, Hiroshima University Hospital, 1-2-3 Kasumi, Minami-ku, Hiroshima, 734-8551 Japan; 2 Department of Gastrointestinal and Hepato-Biliary-Pancreatic Surgery, Nippon Medical School, Tokyo, Japan; 3 Department of Gastroenterology, Nippon Medical School, Graduate School of Medicine, Tokyo, Japan; 4 Department of Medicine, Division of Gastroenterology, Jichi Medical University, Tochigi, Japan; 5 Department of Endoscopic Research and International Education, Jichi Medical University, Tochigi, Japan; 6 Department of Colorectal Surgery, Tokyo Metropolitan Cancer and Infectious Diseases Center Komagome Hospital, Tokyo, Japan; 7 Department of Gastrointestinal and General Surgery, Tokyo Metropolitan Ohkubo Hospital, Tokyo, Japan; 8 Division of Colon and Rectal Surgery, Shizuoka Cancer Center, Shizuoka, Japan; 9 Division of Endoscopy, Shizuoka Cancer Center, Shizuoka, Japan; 10 Department of Gastrointestinal Oncology, Osaka International Cancer Institute, Osaka, Japan; 11 Department of Gastroenterology and Hepatology, Gunma University Graduate School of Medicine, Gunma, Japan; 12 Department of Gastroenterology, National Hospital Organization Kure Medical Center and Chugoku Cancer Center, Hiroshima, Japan; 13 Department of Gastrointestinal Endoscopy and Medicine, Hiroshima University, Hiroshima, Japan; 14 Digestive Disease Center, Showa Medical University Northern Yokohama Hospital, Kanagawa, Japan; 15 Department of Lower Gastrointestinal Medicine, Cancer Institute Hospital of the Japanese Foundation for Cancer Research, Tokyo, Japan; 16 Department of Gastroenterological Surgery, Toranomon Hospital, Tokyo, Japan; 17 Department of Surgery, Kyorin University School of Medicine, Tokyo, Japan; 18 Division of Lower Gastrointestinal Surgery, Department of Surgery, Hyogo Medical University, Hyogo, Japan; 19 Department of Surgery, Institute of Gastroenterology, Tokyo Women’s Medical University, Tokyo, Japan; 20 Department of Surgery, Saiseikai Kazo Hospital, Saitama, Japan; 21 Department of Gastroenterology and Hepatology, Institute of Science Tokyo, Tokyo, Japan; 22 Department of Gastrointestinal Surgery, Institute of Science Tokyo, Tokyo, Japan; 23 Division of Gastroenterology, Department of Internal Medicine, Iwate Medical University, Iwate, Japan; 24 Department of Diagnostic Pathology, Iwate Medical University, Iwate, Japan; 25 Division of Gastroenterology, Matsuyama Red Cross Hospital, Ehime, Japan; 26 Department of Gastroenterological Surgery, Saitama Medical University International Medical Center, Saitama, Japan; 27 Division of Gastrointestinal Surgery, Tokyo Women’s Medical University, Tokyo, Japan; 28 Department of Endoscopy, The Jikei University School of Medicine, Tokyo, Japan; 29 Department of Surgery, Hiroshima City Hiroshima Citizens Hospital, Hiroshima, Japan; 30 Department of Gastroenterology and Hepatology, Kindai University Faculty of Medicine, Osaka, Japan; 31 Department of Gastroenterology and Hepatology, Kawanishi City Medical Center, Hyogo, Japan; 32 Department of Surgery, Kurume University School of Medicine, Fukuoka, Japan; 33 Department of Gastroenterology and Endoscopy, National Cancer Center Hospital East, Chiba, Japan; 34 Department of Gastroenterology, IMSUT Hospital, The Institute of Medical Science, The University of Tokyo, Tokyo, Japan; 35 Department of Colorectal Surgery, National Cancer Center Hospital East, Chiba, Japan; 36 Division of Gastroenterology, Department of Internal Medicine, Faculty of Medicine, Saga University, Saga, Japan; 37 Department of Coloproctological Surgery, Faculty of Medicine, Juntendo University, Tokyo, Japan; 38 Department of Human Pathology, Juntendo University Graduate School of Medicine, Tokyo, Japan; 39 Department of Gastrointestinal Surgery, Kansai Medical University Hospital, Osaka, Japan; 40 Department of Clinical Oncology, Kyoto University Hospital, Kyoto, Japan; 41 Department of Surgery, Teikyo University Chiba Medical Center, Chiba, Japan; 42 Department of Gastroenterology, Maebashi Red Cross Hospital, Gunma, Japan; 43 Department of Gastroenterological Surgery, Aichi Cancer Center Hospital, Aichi, Japan; 44 Department of Gastroenterology, Asahikawa City Hospital, Hokkaido, Japan; 45 Department of Colorectal Surgery, National Cancer Center Hospital, Tokyo, Japan; 46 Endoscopy Division, National Cancer Center Hospital, Tokyo, Japan; 47 Department of Surgery, Coloproctology Center Takano Hospital, Kumamoto, Japan; 48 Department of Surgical Oncology, The University of Tokyo, Tokyo, Japan; 49 Department of Gastroenterology and Oncology, Tokushima University Graduate School of Biomedical Sciences, Tokushima, Japan; 50 Department of Coloproctology, Aizu Medical Center, Fukushima Medical University, Fukushima, Japan; 51 Department of Surgery, National Defense Medical College, Saitama, Japan; 52 Department of Medicine and Clinical Science, Graduate School of Medical Sciences, Kyushu University, Fukuoka, Japan; 53 Department of Colorectal Surgery, Japanese Red Cross Medical Center, Tokyo, Japan; 54 Department of Advanced Endoscopy, Fujita Health University School of Medicine, Aichi, Japan; 55 Department of Surgery, NHO Osaka National Hospital, Osaka, Japan; 56 Division of Digestive Surgery, Department of Surgery, Kyoto Prefectural University of Medicine, Kyoto, Japan; 57 Department of Gastroenterology, Hiroshima City North Medical Center Asa Citizens Hospital, Hiroshima, Japan; 58 Department of Surgery, Japanese Red Cross Omori Hospital, Tokyo, Japan; 59 Institute of Science Tokyo, Tokyo, Japan; 60 Division of Molecular and Diagnostic Pathology, Niigata University Graduate School of Medical and Dental Sciences, Niigata, Japan; 61 Department of Gastroenterology, JA Onomichi General Hospital, Hiroshima, Japan

**Keywords:** Metastatic tumor, Small bowel, Primary tumor, Enteroscopy

## Abstract

**Background:**

Owing to the rarity of metastatic tumors in the small bowel, their clinicopathological features, and prognostic factors remain poorly understood. This study aimed to clarify the clinicopathological features and factors associated with the prognosis of patients with small bowel metastasis from other organs in Japan.

**Methods:**

We retrospectively examined 253 patients who were histopathologically diagnosed with small bowel metastases between January 2008 and December 2017 at multiple institutions in Japan. We identified the clinicopathological features of the condition and determined the factors associated with the prognosis of these patients.

**Results:**

Obstructive symptoms were the most frequent clinical presentations (39% abdominal pain and 18% vomiting), while gastrointestinal bleeding was observed in 27% of patients. The diagnostic modalities included enteroscopy (33%), balloon-assisted enteroscopy (30%), and capsule endoscopy (13%). The most common primary tumor was lung cancer (38%), followed by colorectal cancer (18%), gastric cancer (9%), and malignant melanoma (6%). Surgical intervention, including tumor resection or bypass surgery, was performed in 79% of patients. The cumulative survival rates of patients at 12, 24, and 60 months were 49%, 36%, and 22%, respectively. Multivariate analysis identified surgery as a significant factor for improving overall survival (HR = 0.56, 95% CI 0.35–0.89, *p* = 0.01).

**Conclusions:**

The lung cancer is the most frequent primary tumor of metastatic tumors in the small bowel. Surgical intervention was associated with improved survival outcomes.

**Supplementary Information:**

The online version contains supplementary material available at 10.1007/s00535-025-02322-z.

## Introduction

Although the small bowel constitutes approximately 75% of the digestive tract, small bowel tumors are rare compared with tumors in other gastrointestinal regions. Notably, small bowel tumors account for approximately 3% of all gastrointestinal tract malignancies [1]. The increased use of small bowel endoscopy has led to increased detection of small bowel tumors. A French population-based study reported that the incidence of small bowel malignancies has increased in recent years [2]. The histological types of malignant tumors of the small bowel include adenocarcinomas, malignant lymphomas, neuroendocrine tumors, and gastrointestinal stromal tumors, although the ratio is different over time in the United States [3]. Recent multicenter studies in Japan have further clarified the clinicopathological characteristics of primary small bowel cancer [4, 5]. Metastatic tumors in the small bowel comprise approximately 2–16% of all malignant small bowel tumors [6–11].

These tumors spread via hematogenous metastasis or direct invasion. Hematogenous metastases typically originate from lung cancer, malignant melanoma, and breast cancer, whereas direct invasion is commonly associated with colon and ovarian cancer [12]. Nonetheless, large-scale analyses of primary tumor sites remain limited, and the clinicopathology of metastatic tumors in the small bowel remains unclear. Owing to the rarity of the metastatic tumors in the small bowel, the treatment strategy for this condition is not standardized and depends on the primary tumor. These tumors typically develop in patients with advanced stage disease and poor performance status and can be life-threatening owing to complications such as gastrointestinal bleeding, bowel obstruction, or perforation, necessitating surgical interventions. In recent years, significant advancements in chemotherapy have improved long-term survival in patients with advanced cancer [13–15]. Given these advancements, evaluating the impact of multidisciplinary treatments, including surgery and chemotherapy, on patient prognosis is essential. In this study, we aimed to clarify the clinicopathological features and factors associated with the prognosis of patients with small bowel metastasis from other organs in Japan.

## Methods

**Fig. 1:**
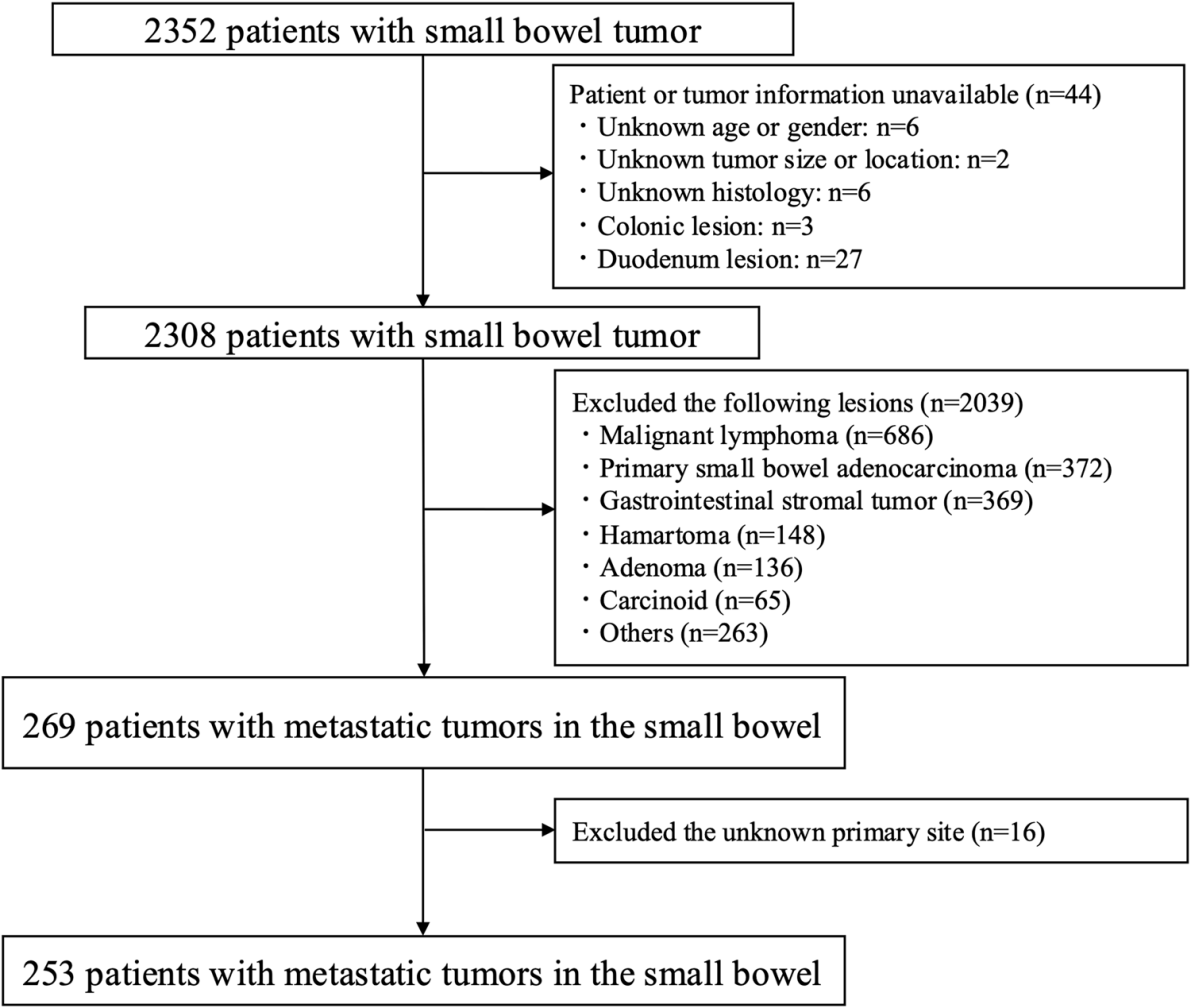
Flowchart for enrolled patients in this study

Figure 1 illustrates the study flowchart. Data were collected from 2352 patients diagnosed with small bowel tumors between January 2008 and December 2017 across 44 institutions affiliated with the Japanese Society for Cancer of the Colon and Rectum (JSCCR). Research on malignant tumors of the small bowel, through collection and analyses of such patients, was initiated by the JSCCR [4, 5]. Of the 2352 patients, we excluded 2039 patients with primary tumors of the small bowel and 44 patients in whom information regarding the tumor was unavailable. In total, 269 had metastatic tumors in the small bowel. Sixteen patients were excluded due to the inability to identify the primary tumor clinicopathologically, leaving a total of 253 patients with metastatic tumors in the small bowel included in the study.

The protocol of this study was approved by the ethics committee of the JSCCR (approval date: August 30, 2019) and each participating institution. This study complied with the ethical standards of the 1964 Declaration of Helsinki and its subsequent amendments.

### Histological evaluation

Each institution conducted pathological assessments of resected specimens or biopsy samples. Metastatic tumors in the small bowel were defined as histologically confirmed secondary tumors involving the small bowel, originating from a non-small bowel primary tumor. Each patient was identified and validated by participating institutions based on pathology reports and clinical data. In principle, metastatic tumors in the small bowel were diagnosed by pathologists at each participating institution. In general, the pathological diagnosis of metastatic small bowel tumors was based on the identification of histological similarity between the small bowel lesion and the primary tumor, supported by immunohistochemical findings. The diagnosis required the presence of malignant epithelial or mesenchymal cells within the small bowel wall without evidence of mucosal origin, continuity with adjacent organs, or features suggestive of primary small bowel cancer. Common immunohistochemical markers were applied as appropriate to confirm the origin of metastasis based on clinical information. We excluded patients in which a clinically or pathologically evident primary site could not be identified. In the present study, patients involving direct invasion from other organ tumors or peritoneal dissemination were excluded.

### Investigated variables

Clinicopathological variables assessed included age, sex, and symptoms at diagnosis, including abdominal pain, vomiting, gastrointestinal bleeding, and intestinal obstruction. Diagnostic modalities, including enteroscopy, such as capsule endoscopy (CE), single-balloon enteroscopy (SBE), and double-balloon enteroscopy (DBE), were also evaluated. Additionally, data on tumor location, tumor number, and treatment method were collected. The prognosis of patients with metastatic tumors in the small bowel was evaluated along with 5-year overall survival (OS). The OS rate was defined as the percentage of patients who survived for a specified duration following diagnosis. Finally, OS rates were compared based on primary tumor (lung cancer vs. other tumors), age, tumor number, and treatment approach (surgery and/or chemotherapy).

### Statistical analysis

All data are presented as percentages and mean ± standard deviation. OS was estimated using the Kaplan–Meier method and differences were analyzed using the log-rank test. Cox regression analysis was performed to calculate hazard ratios (HRs) for OS. A *p* value < 0.05 was considered statistically significant. Missing data were handled using pairwise deletion, in which patients with missing values for a specific variable were excluded only from analyses involving that variable. Therefore, all available data were used for each statistical test, to minimize data loss. Variables with clinical relevance, as well as those with a *p* value < 0.1 in the univariate analysis, were included in the multivariate model to minimize confounding. Previous studies have identified several prognostic factors in primary small bowel cancer, including patient age [16], the presence of symptoms [4], intestinal obstruction [17], surgery [16], and chemotherapy [5, 16]. The JMP statistical software version 18.1.0 (SAS Institute, Cary, NC, USA) was used for all statistical analyses.

## Results

Of the 2352 patients with small bowel tumors registered in the JSCCR database, 269 patients (11%) were diagnosed with metastatic tumors in the small bowel; among these, 16 patients (6%) were excluded from the present analysis owing to a lack of information regarding the location of the primary tumor. Therefore, 253 patients (11%) with metastatic tumors in the small bowel were included in the present analysis. The baseline characteristics of these patients are shown in Table 1.

**Table 1:** Baseline characteristics of the enrolled patients

Variables	Metastatic tumors in the small bowel (*N* = 253)
Age (years), mean (± SD)	65.1 ± 12.5
Sex, male/female	178/75
Chief complaint	
Presence	165 (65)
Absence	55 (22)
Unknown	33 (13)
Abdominal pain	
Presence	99 (39)
Absence	113 (45)
Unknown	41 (16)
Vomiting	
Presence	46 (18)
Absence	160 (63)
Unknown	47 (19)
Intestinal obstruction	
Presence	63 (25)
Absence	153 (60)
Unknown	37 (15)
Gastrointestinal bleeding	
Occult	38 (15)
Overt	30 (12)
Absence	140 (55)
Unknown	45 (18)
Diagnostic modality*	
Enteroscopy	84 (33)
DBE	66 (26)
CE	33 (13)
SBE	9 (4)
EGD	11 (4)
CS	13 (5)
Location	
Jejunum	133 (53)
Ileum	97 (38)
Both	13 (5)
Unknown	10 (4)
Tumor number	
Single	215 (85)
Multiple	28 (11)
Unknown	10 (4)
Treatment	
Surgery	200 (79)
Alone	124 (49)
Combination with chemotherapy	76 (30)
Chemotherapy	28 (11)
Best supportive care	18 (7)
Radiation	1 (0.4)
Endoscopic treatment	1 (0.4)
Unknown	5 (2)

^*^ Duplicated

Data represented as *n* (%) and mean ± SD

*DBE* double balloon endoscopy, *CE* capsule endoscopy, SBE: single balloon endoscopy, *EGD* esophagogastroduodenoscopy, *CS* colonoscopy

Symptoms were present in 65% of patients, with abdominal pain being the most common (39%), followed by gastrointestinal bleeding (27%) and vomiting (18%). Imaging revealed intestinal obstruction in 25% of patients. In asymptomatic patients, metastatic tumors were detected during surveillance imaging examinations or staging work-up for the primary tumor. Regarding diagnostic modalities, small bowel endoscopies were performed in 33% of patients. The most used device was DBE (26%), followed by CE (13%) and SBE (4%). Metastatic tumors were more frequent in the jejunum (53%) than in the ileum (38%), with multiple lesions observed in 11% of patients. For treatment approaches, 79% of the patients underwent surgery, including surgical bypass procedures, while 41% received chemotherapy.

The distribution of the primary tumor sites is shown in Table 2. The most common primary tumors were lung cancer (38%), followed by colorectal cancer (18%), gastric cancer (9%), malignant melanoma (6%), and renal cancer (4%). Table 3 summarizes the metastatic sites and number of lesions in the small bowel, categorized by the location of the primary tumor. Although multiple metastatic lesions were uncommon for all malignancies (11%), malignant melanoma (27%), uterine cancer (20%), breast cancer (20%), and biliary tract cancer (20%) had a relatively high frequency of patients with multiple lesions in the small bowel. Furthermore, although the jejunum was the most frequent site of metastasis in the small bowel overall (53%), the ileum was more commonly affected in patients with metastases from colorectal cancer (59%), ovarian cancer (71%), breast cancer (60%), and biliary tract cancer (60%).

**Table 2:** The breakdown of primary tumor of metastatic tumors in the small bowel

Primary tumor	Metastatic tumors in the small bowel *N* = 253
Lung cancer	95 (38)
Colorectal cancer	46 (18)
Gastric cancer	24 (9)
Malignant melanoma	15 (6)
Renal cancer	11 (4)
Ovarian cancer	7 (3)
Pancreatic cancer	7 (3)
Nasopharyngeal cancer	7 (3)
Uterine cancer	5 (2)
Breast cancer	5 (2)
Biliary tract cancer	5 (2)
Liver cancer	4 (2)
Cervical cancer	3 (1)
Bladder cancer	3 (1)
Others	16 (6)

Data represented as *n* (%)

**Table 3:** Metastatic tumor number and metastatic site of small bowel according to primary tumor with metastatic tumors in the small bowel

Primary tumor	Metastatic tumors, *n* = 253 Single/multiple/unknown
a. Metastatic tumor number	
Overall	215/28/10 (85/11/4)
Lung cancer	82/11/2 (86/12/2)
Colorectal cancer	38/3/5 (83/7/11)
Gastric cancer	22/2/0 (92/8/0)
Malignant melanoma	11/4/0 (73/27/0)
Renal cancer	10/1/0 (91/9/0)
Ovarian cancer	7/0/0 (100/0/0)
Pancreatic cancer	6/0/1 (86/0/14)
Nasopharyngeal cancer	5/1/1 (71/14/14)
Uterine cancer	3/1/1 (60/20/20)
Breast cancer	4/1/0 (80/20/0)
Biliary tract cancer	4/1/0 (80/20/0)
Others	23/3/0 (88/12/0)

Primary tumor	Metastatic tumors, *n* = 253 Jejunum/ileum/both/unknown
b. Metastatic site of small bowel	
Overall	133/97/13/10 (53/38/5/4)
Lung cancer	65/25/3/2 (68/26/3/2)
Colorectal cancer	13/27/1/5 (28/59/2/11)
Gastric cancer	13/9/2/0 (54/38/8/0)
Malignant melanoma	8/5/2/0 (53/33/13/0)
Renal cancer	6/4/1/0 (55/36/9/0)
Ovarian cancer	2/5/0/0 (29/71/0/0)
Pancreatic cancer	6/0/0/1 (86/0/0/14)
Nasopharyngeal cancer	4/2/0/1 (57/29/0/14)
Uterine cancer	2/1/1/1 (40/20/20/20)
Breast cancer	1/3/1/0 (20/60/20/0)
Biliary tract cancer	2/3/0/0 (40/60/0/0)
Others	11/13/2/0 (42/50/8/0)

Data represented as *n* (%)

Table 4 presents the association between primary tumors and symptoms. The primary tumor most likely to cause gastrointestinal bleeding was renal cancer (73%, 8/11), malignant melanoma (47%, 7/15), ovarian cancer (43%, 3/7), and pancreatic cancer (43%, 3/7). In contrast, the primary tumor most likely to cause intestinal obstruction was breast cancer (60%, 3/5), gastric cancer (50%, 12/24), biliary tract cancer (40%, 2/5), and ovarian cancer (29%, 2/7).

**Table 4:** Frequency of symptoms according to primary site with metastatic tumors in the small bowel

Primary tumor	Metastatic tumors, *n* = 253 Bleeding (+)/bleeding (-)/unknown
a. Gastrointestinal bleeding	
Overall	68/140/45 (27/55/18)
Renal cancer	8/2/1 (73/18/9)
Malignant melanoma	7/7/1 (47/47/7)
Ovarian cancer	3/3/1 (43/43/14)
Pancreatic cancer	3/3/1 (43/43/14)
Uterine cancer	2/2/1 (40/40/20)
Lung cancer	24/56/15 (25/59/16)
Colorectal cancer	7/23/16 (15/50/35)
Breast cancer	1/4/0 (20/80/0)
Biliary tract cancer	1/4/0 (20/80/0)
Gastric cancer	2/21/1 (8/88/4)
Nasopharyngeal cancer	0/3/4 (0/43/57)
Others	10/12/4 (38/46/15)

Primary tumor	Metastatic tumors, *n* = 253 Obstruction (+)/obstruction (−)/unknown
b. Intestinal obstruction	
Overall	63/153/37 (25/60/15)
Breast cancer	3/2/0 (60/40/0)
Gastric cancer	12/11/1 (50/46/4)
Biliary tract cancer	2/3/0 (40/60/0)
Ovarian cancer	2/4/1 (29/57/14)
Lung cancer	26/60/9 (27/63/9)
Colorectal cancer	9/23/14 (20/50/30)
Uterine cancer	1/3/1 (20/60/20)
Renal cancer	2/9/0 (18/82/0)
Malignant melanoma	1/13/1 (7/87/7)
Nasopharyngeal cancer	0/3/4 (0/43/57)
Pancreatic cancer	0/5/2 (0/71/29)
Others	5/17/4 (19/65/15)

Data represented as *n* (%)

**Fig. 2:**
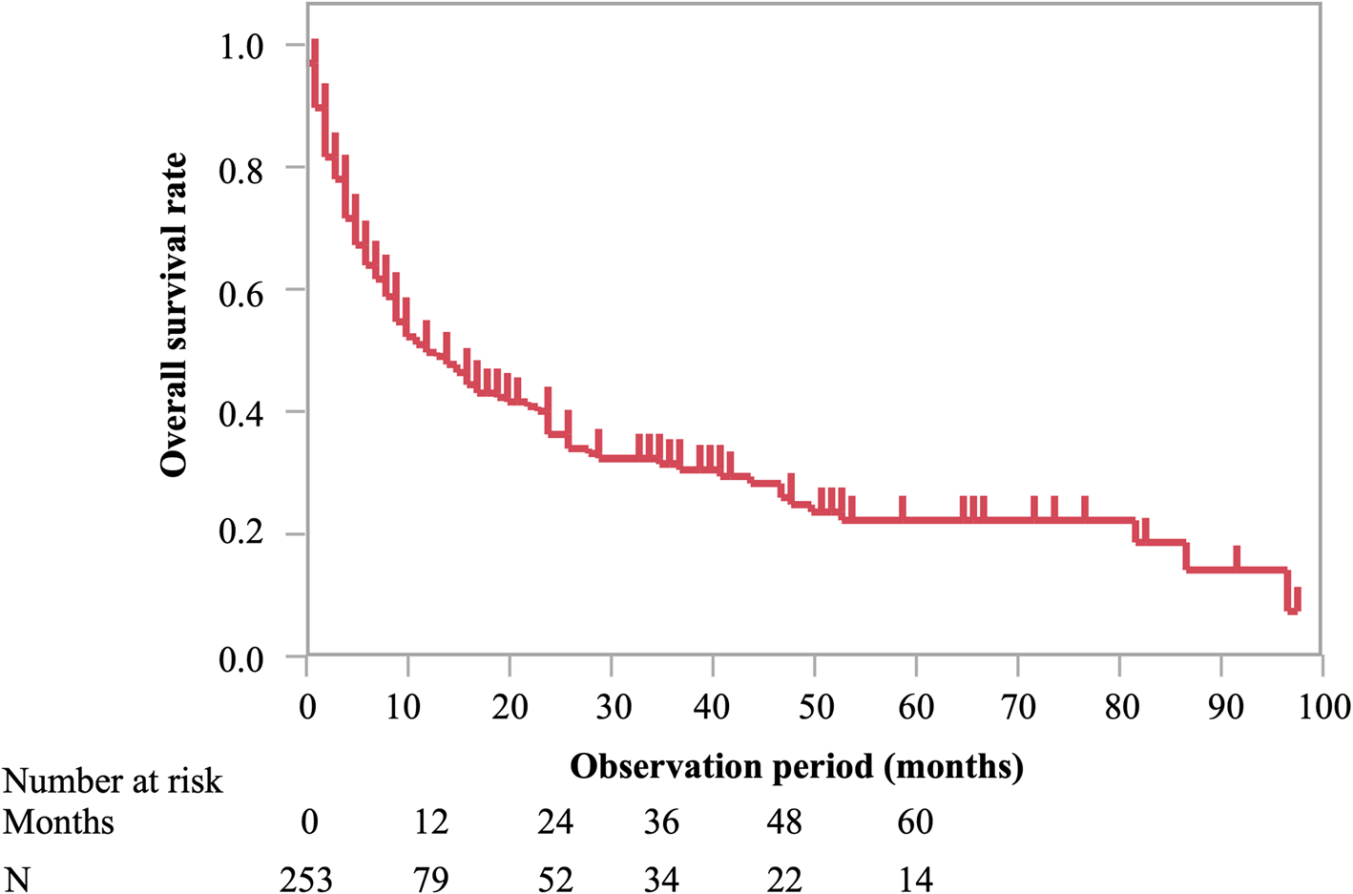
Analysis of overall survival (OS) in patients with metastatic tumors in the small bowel. The cumulative survival rates of metastatic tumors in the small bowel at 12, 24, and 60 months were 49%, 36%, and 22%, respectively

**Fig. 3:**
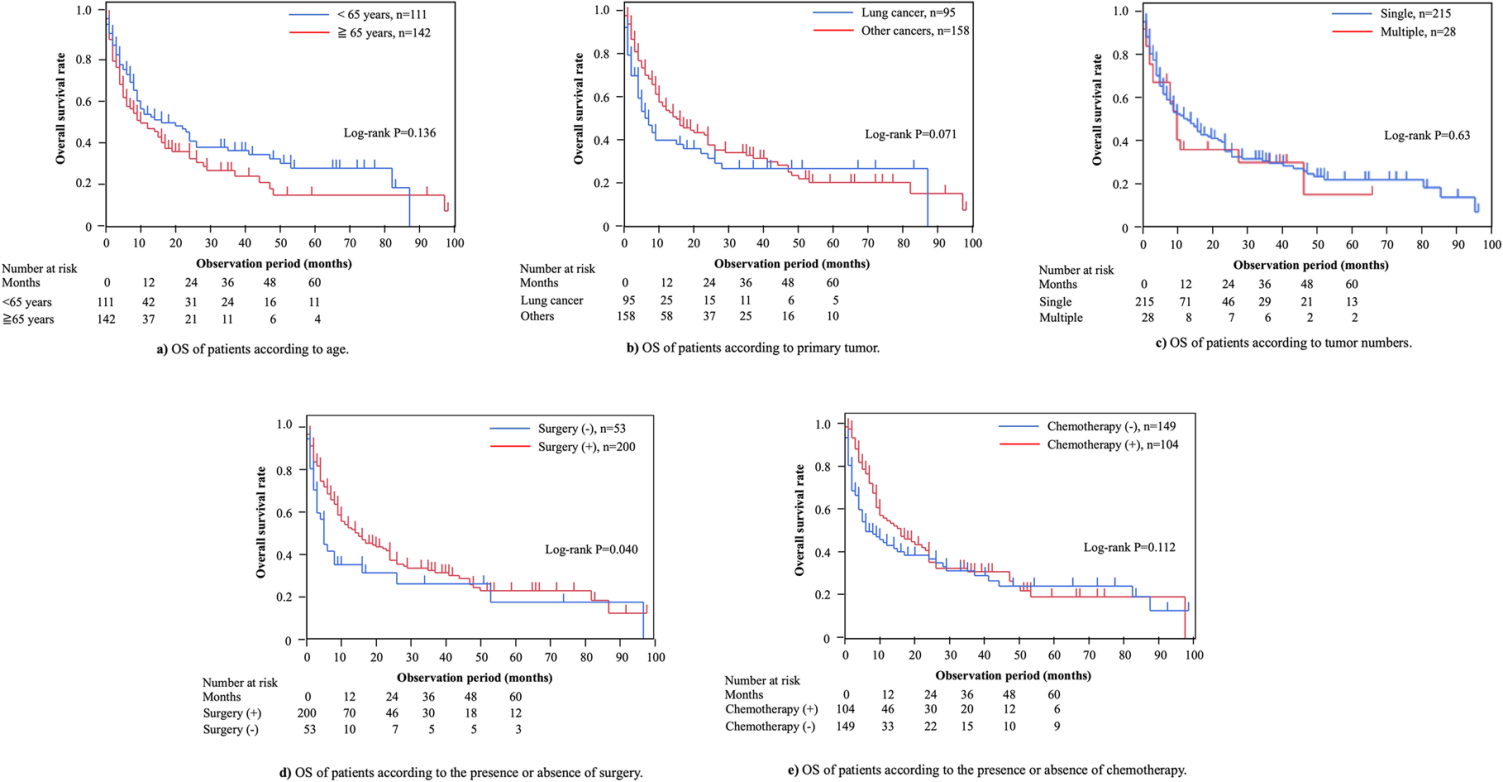
Analysis of overall survival (OS) in patients with metastatic tumors in the small bowel according to various factors. **a** OS of patients according to age. **b** OS of patients according to primary tumor; **c** OS of patients according to the presence or absence of symptoms; **d** OS of patients according to the presence or absence of surgery; **e** OS of patients according to the presence or absence of chemotherapy. **a** The cumulative survival rates of patients aged < 65 years were 53%, 40%, and 27% at 12, 24, and 60 months, respectively. In contrast, for patients aged ≥ 65 years, the cumulative survival rates were 46%, 32%, and 15% at the same time points (*p* = 0.14, log-rank test). **b** The cumulative survival rates of patients with metastatic tumors in the small bowel from lung cancer were 40%, 32%, and 27% at 12, 24, and 60 months. In contrast, for patients with metastatic tumors in the small bowel from non-lung cancer, the cumulative survival rates were 54%, 38%, and 21% at the same time points (*p* = 0.07, log-rank test). **c** The respective cumulative survival rates of patients with multiple metastatic tumors in the small bowel were 36%, 36%, and 15% at 12, 24, and 60 months. In contrast, in patients with a single metastatic tumor in the small bowel the respective cumulative survival rates were 51%, 35%, and 22% at the same time points. **d** The cumulative survival rates of patients who underwent surgery were 52%, 37%, and 23% at 12, 24, and 60 months, respectively. In contrast, in patients who did not undergo surgery, the cumulative survival rates were 35%, 31%, and 26% at the same time points, respectively (*p* = 0.04, log-rank test). **e** The cumulative survival rates of patients who received chemotherapy were 55%, 36%, and 20% at 12, 24, and 60 months, respectively. In contrast, in patients who did not receive chemotherapy, the cumulative survival rates were 44%, 37%, and 25% at the same time points, respectively (*p* = 0.11, log-rank test)

The Kaplan–Meier curves for OS in patients with metastatic tumors in the small bowel are shown in Fig. 2. The cumulative OS rates at 12, 24, and 60 months were 49%, 36%, and 22%, respectively. Supplementary Fig. 1 shows the OS of patients with metastatic tumors in the small bowel, according to the primary tumor location and limited to those with more than 10 patients. The cumulative OS rates at 12, 24, and 60 months for patients with metastatic tumors in the small bowel were 40%, 34%, and 27% from lung cancer; 80%, 61%, and 27% from colorectal cancer; 49%, 24%, and 24% from gastric cancer; 51%, 42%, and 28% from malignant melanoma; and 57%, 57%, and 23% from renal cancer, respectively. Figure 3 shows Kaplan–Meier curves of OS according to age (≥ 65 or < 65 years; Fig. 3a), the primary tumor (lung cancer or other tumors; Fig. 3b), the number of metastatic tumors (multiple or single; Fig. 3c), surgical intervention (Fig. 3d), and chemotherapy (Fig. 3e). Although no significant differences in OS were observed across the different primary tumors, age, tumor number, and presence or absence of chemotherapy groups, OS was significantly better in patients with metastatic tumors in the small bowel who underwent surgery than in those who did not. Table 5 summarizes the univariate and multivariate analyses of prognostic factors for OS. Surgery was significantly associated with prolonged OS in univariate analysis (HR = 0.66, 95% confidence interval [CI]: 0.43–0.99, *p* < 0.05). Multivariate analysis confirmed that surgery was the only independent factor associated with improved OS (HR = 0.56, 95% CI: 0.35–0.89, *p* = 0.01).

**Table 5:** Cox regression analysis for overall survival in patients with metastatic tumors in the small bowel (*n* = 269)

Variables		*N*	Univariate analysis		Multivariate analysis	
			HR (95%CI)	*p* Value	HR (95%CI)	*p* Value
Sex	Male	178	1.14 (0.77–1.70)	0.50	1.13 (0.73–1.75)	0.58
	Female	75	Ref		Ref	
Age	≧65	142	1.29 (0.91–1.82)	0.15	1.38 (0.95–2.02)	0.09
	< 65	111	Ref		Ref	
Primary tumor	Lung cancer	95	1.37 (0.96–1.95)	0.08	1.34 (0.90–1.99)	0.15
	Other tumors	158	Ref		Ref	
Tumor number	Multiple	28	1.13 (0.68–1.88)	0.64	1.42 (0.77–2.61)	0.26
	Single	215	Ref		Ref	
Gastrointestinal bleeding	Presence	68	1.12 (0.76–1.64)	0.58	1.09 (0.74–1.62)	0.66
	Absence	140	Ref		Ref	
Intestinal obstruction	Presence	63	1.11 (0.75–1.63)	0.60	1.21 (0.78–1.85)	0.39
	Absence	153	Ref		Ref	
Surgery	Presence	200	0.66 (0.43–0.99)	< 0.05	0.56 (0.35–0.89)	0.01
	Absence	53	Ref		Ref	
Chemotherapy	Presence	104	0.77 (0.54–1.07)	0.12	0.71 (0.49–1.03)	0.07
	Absence	149	Ref			

*HR* hazard ratio, *CI* confidence interval, *Ref* reference

## Discussion

Our study revealed that lung cancer (38%), was the most common primary tumor to exhibit metastatic tumors in the small bowel among Japanese patients followed by colorectal cancer (18%), gastric cancer (9%), and malignant melanoma (6%). Metastatic tumors in the small bowel from renal cancer, malignant melanoma, ovarian cancer, and pancreatic cancer frequently present with gastrointestinal bleeding, whereas those originating from breast, gastric, biliary tract, and ovarian cancers often manifest with symptoms of intestinal obstruction. In contrast, metastatic tumors in the small bowel from lung cancer tend to cause gastrointestinal bleeding and intestinal obstruction with comparable frequency. Predicting the clinical manifestations of metastatic tumors in the small bowel based on the primary tumor type may, therefore, aid the optimization of patient management. In this study, surgery was the only independent factor associated with the prolonged survival of patients with metastatic tumors in the small bowel. To the best of our knowledge, this is the first large-scale study to investigate the clinical characteristics and prognosis of metastatic tumors in the small bowel.

The incidence of small bowel tumors has increased in recent years [2]. Metastatic tumors in the small bowel are generally more prevalent than primary adenocarcinomas in Western countries [18]. In the present study, 253 patients with metastatic tumors in the small bowel were identified, as opposed to 372 patients with primary small bowel adenocarcinomas. This may be attributed to racial differences and the exclusion of patients with direct invasion due to peritoneal dissemination. According to previous reports from Germany, malignant melanoma, breast cancer, and pancreatic cancer are the most common primary tumors to metastasize to the gastrointestinal tract [19]. Gilg et al. [20] reported that among gastrointestinal metastases, the small bowel was the second most common metastatic site after the stomach. They also reported that malignant melanoma, breast cancer, and pancreatic cancer were the leading causes of upper gastrointestinal metastasis, including those in the small bowel [20]. Additionally, in the United States, lung cancer, malignant melanoma, renal cancer, and breast cancer have been reported to metastasize to the small bowel [1, 21–23]. A study involving a small cohort of patients from Japan identified lung cancer as the most common primary site, aligning with previous reports [24]. Our findings confirm that lung cancer is the most common primary tumor to exhibit metastatic tumors in the small bowel. However, unlike previous studies, we observed a higher incidence of metastases from gastric and colorectal cancers than from malignant melanoma or renal cancer. Gastric cancer is one of the most prevalent cancers in East Asia, including Japan [25]. Although the incidence of colorectal cancer has declined in the United States in recent years, it remains one of the most common cancers in Japan [26]. By contrast, malignant melanoma is less prevalent in Japan than in Western countries [27]. These differences may be attributed to the variations in cancer incidence across racial and ethnic groups. Indeed, the incidence of each cancer type is known to vary by country and ethnicity worldwide. For example, in Japan, the number of cancer patients in 2024 was approximately 979,000. Among men, the most common cancer was prostate cancer (91,800 patients; 16%), followed by colorectal cancer (85,600; 15%), lung cancer (85,000; 15%), gastric cancer (78,900; 14%), and liver cancer (25,000; 4%). Among women, the most common cancer was breast cancer (91,100 patients; 22%), followed by colorectal cancer (67,600; 16%), gastric cancer (41,200; 10%), lung cancer (36,200; 9%), and uterine cancer (28,300; 7%) [28]. In the United States, the projected number of new cancer patients in 2025 is approximately 2,041,910 [29]. In terms of the estimated number of cancer patients in 2025, the most common cancer among men in the United States is prostate cancer (313,780 patients; 30%), followed by lung cancer (110,680; 11%), colorectal cancer (82,460; 8%), bladder cancer (65,080; 6%), and melanoma (60,550; 6%). Among women, the most common cancer is breast cancer (316,950; 32%), followed by lung cancer (115,970; 12%), colorectal cancer (71,810; 7%), uterine cancer (69,120; 7%), and melanoma (44,410; 4%) [29]. Thus, the epidemiology of cancer varies widely across the world, and this difference in cancer epidemiology may have contributed to the observed findings in the present study. Conversely, because cancer incidence varies across countries, data collected from multiple nations may be subject to inconsistencies. By compiling data exclusively from a single country, such inconsistencies can be avoided, thereby highlighting the rarity of this study.

In our study, multivariate analysis revealed that surgery was the only independent factor associated with improved prognosis in patients with metastatic tumors in the small bowel. These tumors can further reduce life expectancy owing to gastrointestinal complications, such as bowel obstruction and gastrointestinal bleeding. Surgery may effectively alleviate these symptoms and improve patient outcomes [30]. Previous reports have also shown that surgical resection of metastases improves OS in patients with metastatic tumors in the small bowel from malignant melanoma [31–33]. Additionally, bowel obstruction caused by peritoneal dissemination is often associated with poor OS making surgery unfeasible in certain patients. In such patients, conservative management, including bowel rest, fasting, and intravenous nutrition, is the preferred approach. However, Chouhan et al. [34] reported that in patients of bowel obstruction due to peritoneal dissemination, a combination of transvenous nutrition and chemotherapy is not recommended due to high mortality rates. Therefore, multidisciplinary treatment may not always be the optimal approach, and palliative care should be considered for patients who are not surgical candidates. Endoscopic self-expandable stenting for intestinal obstruction [35] and endoscopic resection for hemorrhagic metastatic lesions [36] may also be considered for symptom management in inoperable patients.

In this study, the presence or absence of chemotherapy had no impact on prognosis. Generally, chemotherapy regimens for patients with advanced cancer and metastases to other organs are comparable to those for primary tumors. Surgery may be required to manage intestinal obstruction or bleeding in patients with small bowel metastasis. However, chemotherapy must be discontinued temporarily if surgery is performed. In our study, 22% of the patients were asymptomatic, and chemotherapy is generally prioritized for such patients. Given the variability in treatment strategies across institutions, establishing standardized criteria for selecting surgery or chemotherapy is essential.

For metastatic tumors in the small bowel, a major concern is the occurrence of gastrointestinal complications, such as bleeding, obstruction, intussusception, and perforation. This study identified gastrointestinal bleeding and intestinal obstruction as the predominant symptoms for the patients with metastatic tumors in the small bowel. Gastrointestinal bleeding was more common in patients with renal cancer (73%) and malignant melanoma (47%) as primary tumors. These cancer types are generally hypervascular [37, 38], suggesting that the characteristics of the primary tumors may have contributed to the signs exhibited by the metastatic lesion. A multicenter retrospective study in France on gastrointestinal metastasis from renal cell carcinoma reported that 70% of the patients presented with anemia and gastrointestinal bleeding [37], which is consistent with our findings. Conversely, patients with breast cancer (60%), gastric cancer (50%), biliary tract cancer (40%), or ovarian cancer (29%) were more likely to have intestinal obstruction [39]. Intestinal obstruction was more frequently observed in patients with primary abdominal tumors, which tended to spread peritoneally. Patients with lung cancer were more prone to gastrointestinal bleeding (25%) and intestinal obstruction (27%). Subjective symptoms are also important factors in identifying the primary site.

This study has some limitations. First, as a retrospective study, it carries an inherent risk of selection bias, particularly when comparing surgical and non-surgical treatment groups. Patients who underwent surgery may have had better overall health or less aggressive disease, potentially influencing survival outcomes. Although we adjusted for confounding factors, residual bias cannot be completely excluded. Second, the study lacked comprehensive details on the types of surgery performed (e.g., segmental resection and bypass surgery) and their associated postoperative outcomes. The absence of these data limits our ability to assess the true prognostic impact of the different surgical approaches. Further prospective multicenter studies with standardized data collection, including detailed surgical and oncological outcomes, are needed to validate our findings and refine treatment strategies for metastatic tumors in the small bowel. Third, the histology of the primary tumors was not analyzed. For instance, lung cancer is classified into non-small-cell carcinoma and small-cell carcinoma, which require distinct management approaches. Notably, non-small-cell carcinoma frequently metastasizes to the small bowel [11, 40]. Future studies with larger datasets are warranted, to further elucidate the histological types of primary tumors involved, and their associated clinical implications. Fourth, endoscopic evaluation was not performed in all patients. Given the historical context of this long-term retrospective study, the number of patients amenable to endoscopic evaluation using CE or BAE was limited. CE is a minimally invasive modality and is suitable for patients with poor general condition. However, in patients with metastatic small bowel tumors, there is a risk of capsule retention, necessitating careful consideration of its use. Moreover, large lesions may not be fully visualized, which can complicate lesion recognition [41]. Additionally, metastatic gastrointestinal tumors often exhibit submucosal tumor-like morphology [20], potentially leading to false-negative findings with CE [11]. Therefore, the indications for small bowel endoscopy should be carefully evaluated based on the patient’s clinical condition and diagnostic necessity. Fifth, the current study spanned approximately 10 years, raising the possibility of temporal bias in the results. In particular, recent advances in chemotherapy, including the development of novel therapies such as immune checkpoint inhibitors, have markedly improved the prognoses of various cancers. Although the presence or absence of chemotherapy was not associated with improved prognosis in the present analysis, it is possible that chemotherapy may contribute to better outcomes in future studies. Sixth, because the study focused exclusively on malignant tumors of the small bowel, the presence or absence of synchronous metastases to other parts of the gastrointestinal tract could not be analyzed. Gilg et al. [20] reported that among metastatic tumors of the gastrointestinal tract, the stomach was the most frequent site, followed by the small bowel (including the duodenum) and then the large intestine. This suggests that some patients in the present study may have had concurrent metastases in other regions of the gastrointestinal tract. The presence of metastases at other sites can influence therapeutic decision-making, and should be further investigated in future studies. Finally, treatment methods vary across institutions, and no consensus exists on optimal treatment strategies. Further large-scale studies to make up for the lacking information in this study are needed to establish standardized management approaches for metastatic tumors in the small bowel.

In conclusion, the prognosis of patients with metastatic tumors in the small bowel is poor, regardless of the primary cancer. Lung cancer is the most common primary tumor to exhibit metastatic tumors in the small bowel. Surgery was associated with improved OS. Symptoms vary depending on the type of primary tumor. Thus, our findings may guide clinicians in selecting diagnostic strategies and surgical indications based on presenting symptoms and primary tumor type. Small-bowel endoscopies were performed in 33% of the patients as a diagnostic modality. Widespread use of this modality will lead to more frequent accurate diagnoses of such lesions.

## Supplementary Information

Below is the link to the electronic supplementary material.

Supplementary file1 (TIFF 35159 KB) (a) Lung cancer. The cumulative OS rates at 12, 24, and 60 months for metastatic tumors in the small bowel originating from lung cancer are 40%, 34%, and 27%, respectively

Supplementary file2 (TIFF 35159 KB)(b) Colorectal cancer. The cumulative OS rates at 12, 24, and 60 months for metastatic tumors in the small bowel originating from colorectal cancer are 80%, 61%, and 27%, respectively

Supplementary file3 (TIFF 35159 KB) (c) Gastric cancer. The cumulative OS rates at 12, 24, and 60 months for metastatic tumors in the small bowel originating from gastric cancer are 49%, 24%, and 24%, respectively

Supplementary file4 (TIFF 35159 KB) (d) Malignant melanoma. The cumulative OS rates at 12, 24, and 60 months for metastatic tumors in the small bowel originating from malignant melanoma are 51%, 42%, and 28%, respectively

Supplementary file5 (TIFF 35159 KB) (e) Renal cancer. The cumulative OS rates at 12, 24, and 60 months for metastatic tumors in the small bowel originating from renal cancer are 57%, 57%, and 23%, respectively
