# Supplementary figures and images for: Clinicopathological features and prognosis of metastatic tumors in the small bowel: a large multicenter analysis of the JSCCR database in Japan
Source: J Gastroenterol. 2025 Nov 18;61(3):266–78. doi: 10.1007/s00535-025-02322-z (PMC12987839; doi:10.1007/s00535-025-02322-z)

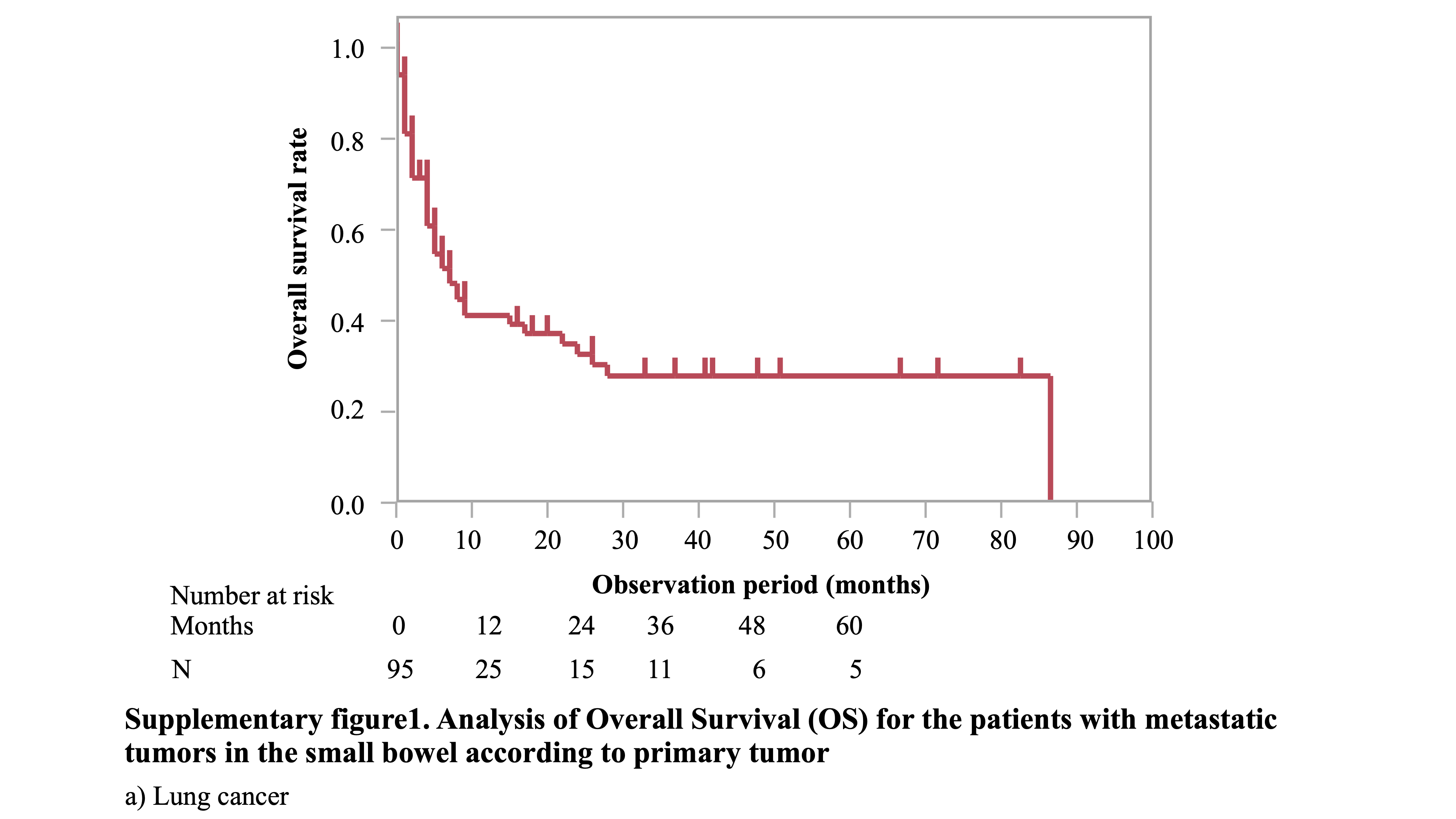

Supplement: Supplementary file 1 — Supplementary file1 (TIFF 35159 KB) (a) Lung cancer. The cumulative OS rates at 12, 24, and 60 months for metastatic tumors in the small bowel originating from lung cancer are 40%, 34%, and 27%, respectively [file 535_2025_2322_MOESM1_ESM.tiff]

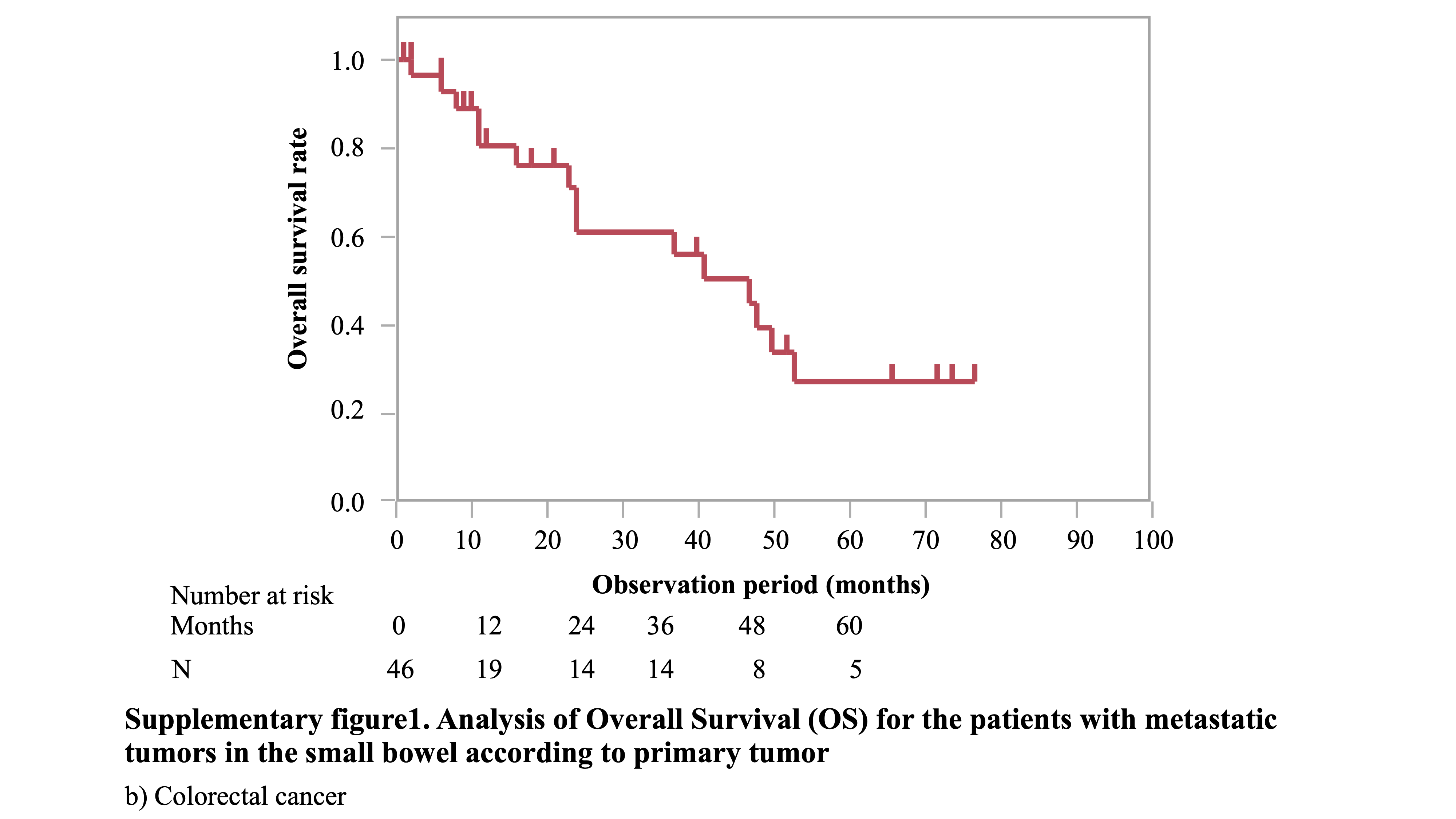

Supplement: Supplementary file 2 — Supplementary file2 (TIFF 35159 KB)(b) Colorectal cancer. The cumulative OS rates at 12, 24, and 60 months for metastatic tumors in the small bowel originating from colorectal cancer are 80%, 61%, and 27%, respectively [file 535_2025_2322_MOESM2_ESM.tiff]

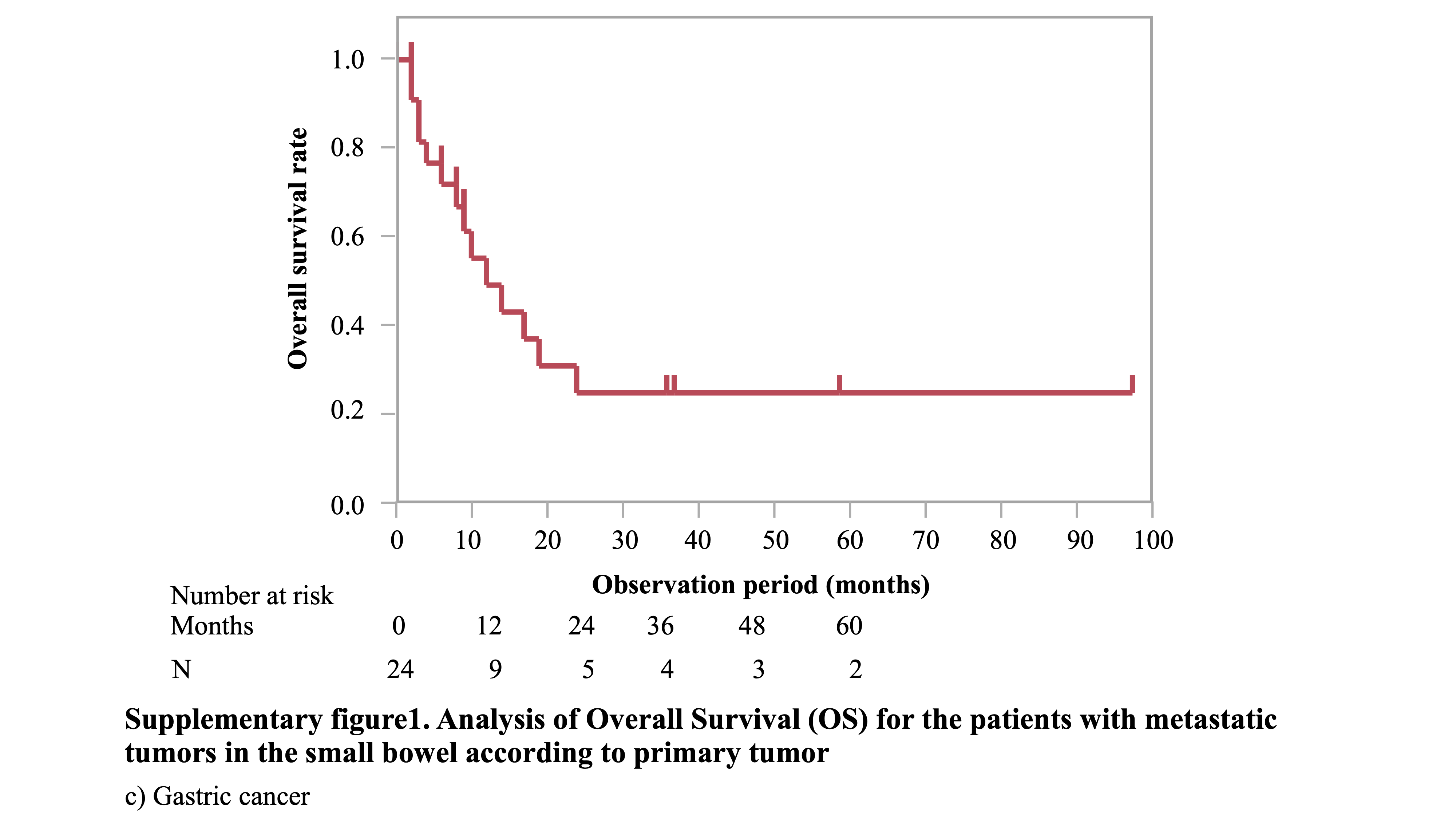

Supplement: Supplementary file 3 — Supplementary file3 (TIFF 35159 KB) (c) Gastric cancer. The cumulative OS rates at 12, 24, and 60 months for metastatic tumors in the small bowel originating from gastric cancer are 49%, 24%, and 24%, respectively [file 535_2025_2322_MOESM3_ESM.tiff]

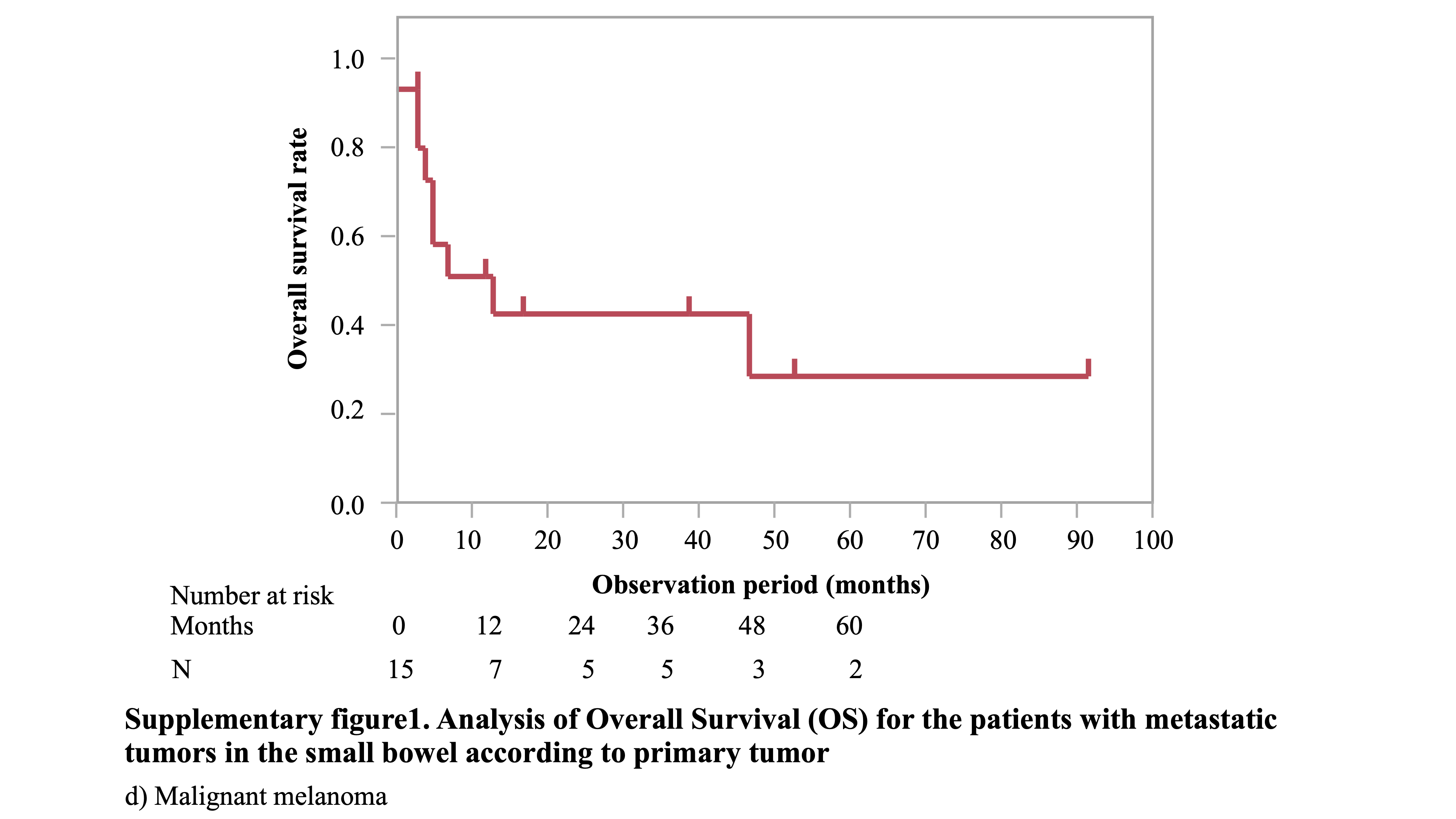

Supplement: Supplementary file 4 — Supplementary file4 (TIFF 35159 KB) (d) Malignant melanoma. The cumulative OS rates at 12, 24, and 60 months for metastatic tumors in the small bowel originating from malignant melanoma are 51%, 42%, and 28%, respectively [file 535_2025_2322_MOESM4_ESM.tiff]

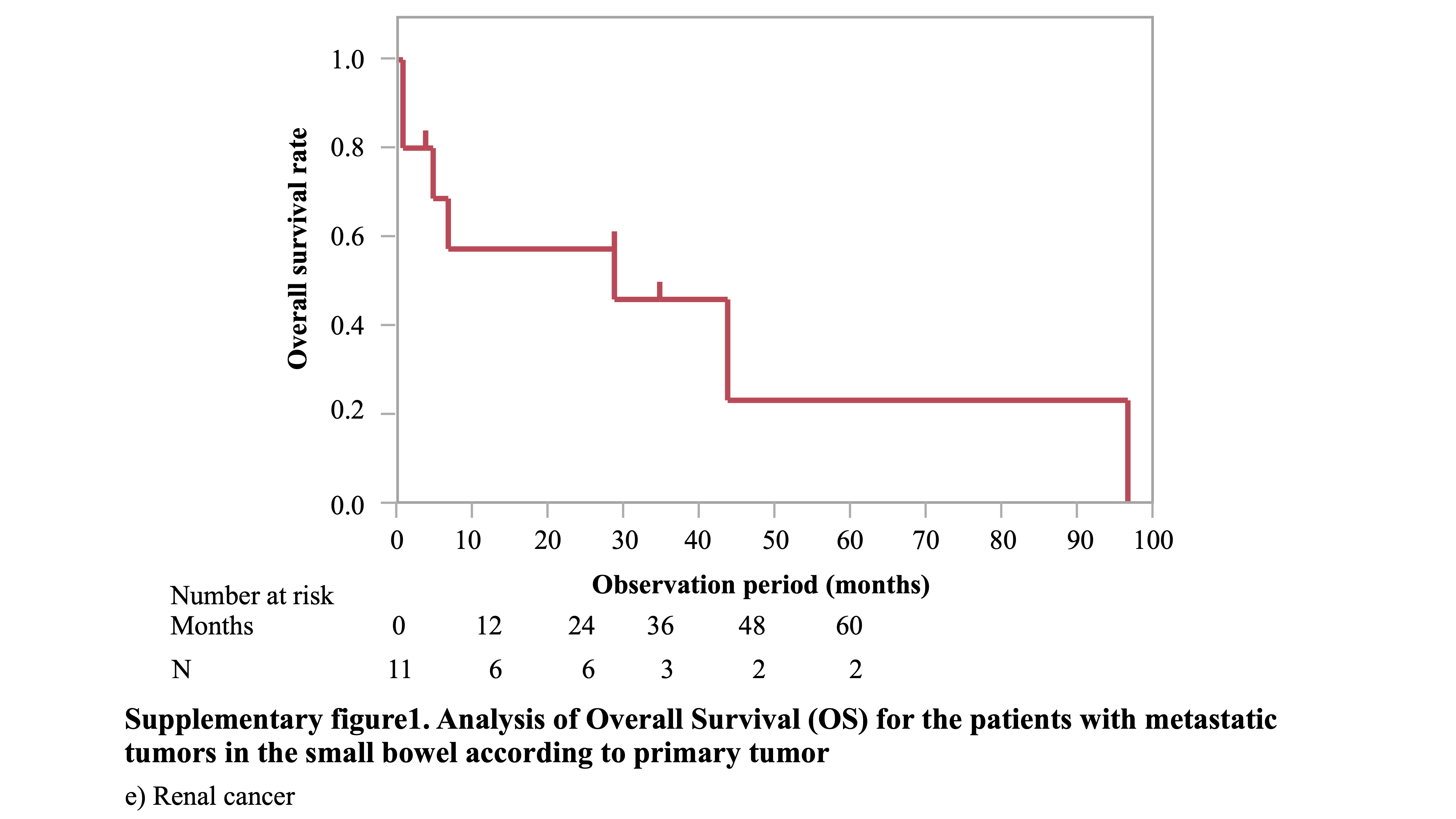

Supplement: Supplementary file 5 — Supplementary file5 (TIFF 35159 KB) (e) Renal cancer. The cumulative OS rates at 12, 24, and 60 months for metastatic tumors in the small bowel originating from renal cancer are 57%, 57%, and 23%, respectively [file 535_2025_2322_MOESM5_ESM.tiff]
